# Spatiotemporal Approaches for Quality Control and Error Correction of Atmospheric Data through Machine Learning

**DOI:** 10.1155/2020/7980434

**Published:** 2020-03-11

**Authors:** Hye-Jin Kim, Sung Min Park, Byung Jin Choi, Seung-Hyun Moon, Yong-Hyuk Kim

**Affiliations:** ^1^Department of Computer Science, Kwangwoon University, 20 Kwangwoon-ro, Nowon-gu, Seoul 01897, Republic of Korea; ^2^R&D Center, Jubix Co., Ltd., B-808, Gunpo IT Valley, 17, Gosan-ro 148beon-gil, Gunpo-si, Gyeonggi-do 15850, Republic of Korea; ^3^Department of Computer Science & Engineering, Seoul National University, 1 Gwanak-ro, Gwanak-gu, Seoul 08826, Republic of Korea

## Abstract

We propose three quality control (QC) techniques using machine learning that depend on the type of input data used for training. These include QC based on time series of a single weather element, QC based on time series in conjunction with other weather elements, and QC using spatiotemporal characteristics. We performed machine learning-based QC on each weather element of atmospheric data, such as temperature, acquired from seven types of IoT sensors and applied machine learning algorithms, such as support vector regression, on data with errors to make meaningful estimates from them. By using the root mean squared error (RMSE), we evaluated the performance of the proposed techniques. As a result, the QC done in conjunction with other weather elements had 0.14% lower RMSE on average than QC conducted with only a single weather element. In the case of QC with spatiotemporal characteristic considerations, the QC done via training with AWS data showed performance with 17% lower RMSE than QC done with only raw data.

## 1. Introduction

Atmospheric data collected in real time are used in many applications for various purposes such as healthcare [1] and combatting natural disasters [2]. For this reason, the stability of collected atmospheric data is of high importance. Additionally, as the collected raw data have a large influence on their analysis, research is needed to ensure stability of the atmospheric data. One of the key factors for stable atmospheric data is error data. Error data can occur for a variety of reasons such as malfunctioning equipment, network issues, or communication issues, all of which can have a major impact on data analysis results. For these reasons, quality control (QC) is used to determine error data. In addition, interpolation is used to accordingly replace error data with more accurate data as needed. To interpolate more accurately than traditional methods such as linear interpolation, there is an approach based on machine learning [3–9].

The studies on time series data using machine learning have been largely conducted for forecasting weather and stock price [10–14]. There are several prior studies related to the various applications of atmospheric data which commonly use machine learning approaches. As a primary example, Cortez and Morais [15] developed a system for predicting forest fires via meteorological data. In that study, a data mining approach was used to predict forest fire outbreaks. Using five methods, including support vector machine (SVM) and random forest, they were able to predict fires through data mining applied to meteorological data acquired from an observation post in the northeast region of Portugal. Spatial correction through machine learning is a subject of active research [4–6]. Kim et al. [4] proposed a correction method for air pressure data acquired from microelectromechanical pressure sensors embedded in smartphones. For the correction method, linear regression was used and correction was performed based on QC conducted on spatial location, temperature, humidity, and individual users. To overcome the shortcomings of standard automatic weather stations (AWSs), Ha et al. [5] constructed a small mobile mini-AWS. Mini-AWSs have advantageous installation, operation, and maintenance costs, as well as reduced installation space requirements. However, correction is needed as the installation area may be affected by the external environment. For this purpose, they employed machine learning for correction of the air pressure data. Kim et al. [6] performed clustering and regression on air pressure data obtained from smartphones by classifying them according to the time domain. Furthermore, they analyzed the results of machine learning methods such as multilayer perceptron (MLP) and support vector regression (SVR). When regression was analyzed with expectation maximization clustering, the mean absolute error of SVR was 26% lower on average than results obtained without the analysis process. Lee et al. [7] were the first to attempt correction using time series data. They conducted research on correcting abnormal data collected by AWS. Using machine learning as a basis, three estimation models (decision tree, artificial neural network, and SVR) were proposed and compared to existing estimation and interpolation methods. Within 5 years and over 692 locations in South Korea, they found that it was better at estimating reference values than existing methods. Kim and Kim [8] proposed the recurrent neural network model for correction of error in drifter data observations. Through experimentation, approximately 14% of the data was corrected and the performance of drifter moving prediction was improved by about 1.4%. Lee et al. [9] were the first to attempt spatial QC using machine learning. In their study, SVR was used to detect and correct abnormal values in observations. Testing with a real-time dataset showed the method utilizing SVR had a 45% lower root mean squared error (RMSE) on average than baseline estimators.

As these examples show, the use of machine learning for atmospheric data research has been active. In particular, there were many studies using machine learning for spatial QC and correction. In this study, we conduct spatiotemporal QC based on machine learning using multipoint time series data and we propose three QC techniques that depend on particular types of training data. The first technique, machine learning-based QC using homogeneous temporal data (MLQC-HT), is the prediction of the current observed value for a given weather element by using data from the past 10 minutes for training. The second technique, machine learning-based QC using nonhomogeneous temporal data (MLQC-NT), is the prediction of the current observed value for a given weather element by using data from the past 10 minutes in the same weather element, as well as current values from other weather elements. Finally, machine learning-based QC using spatiotemporal data (MLQC-ST) is independent of the previous two methods as it is the machine learning of data from sensors in separate locations in the same time instance by using spatiotemporal characteristics. Through these three techniques based on machine learning, we conducted QC, and raw data errors detected by machine learning estimates were corrected.

The remainder of this paper is organized as follows: first, in Section 2, the sensors and atmospheric data used for this study are introduced. In addition, this section describes the preprocessing and basic QC conducted for the machine learning-based QC. In Section 3, the machine learning techniques we used, such as SVR [16], decision table (DT) [17], and MLP [18], as well as the QC methods depending on data types such as MLQC-HT, MLQC-NT, and MLQC-ST, are each described in subsections.  Section 4 explains the experimental methods and environment, and this section describes the experimental results according to the applied QC method. Finally, Section 5 summarizes the conclusions of this study and proposes potential following studies.

## 2. Meteorological Data

### 2.1. IoT Sensors

**Figure 1:**
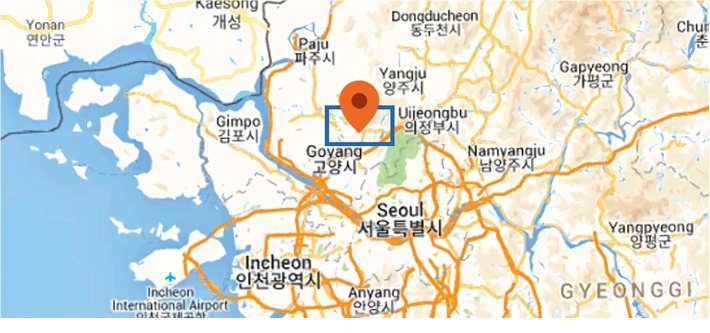
Location of the installed IoT sensors (latitude: 37.708, longitude: 126.895) from Google Maps (https://www.google.com/maps).

We gathered minutely data for seven days between 00 : 30 April 2^nd^ and 24 : 00 April 8^th^ in 2018, from seven different IoT sensors located in Deokyang-gu, Goyang-si, Gyeonggi-do, South Korea (latitude: 37.708, longitude: 126.895) as shown in Figure 1. The types of IoT sensors used and the observed weather element are shown in Table 1, and the sensors were all installed at the same time in December 9, 2016. The collected observation data were preprocessed depending on the sensor type. In the case of solar radiation because it was culminative data, it was converted to instantaneous data in the unit of minutes. Additionally, wind direction (*θ*) and wind velocity ($\left\|\overset{⟶}{W}\right\|$) data, where $\overset{⟶}{W}$ is the wind vector, were converted to *u* and *v*, respectively. The conversion of *u* and *v* is as shown in the following equation:

(1) $$\begin{array}{c} u=\left\|\overset{⟶}{W}\right\|\sin\;\theta ,\\ v=\left\|\overset{⟶}{W}\right\|\cos\;\theta . \end{array}$$

### 2.2. Basic QC

On the collected meteorological data, obviously false data were filtered through basic QCs to increase the possibility that the filtered data are true. Basic QC was conducted sequentially to immediately detect abnormal data obtained by sensors. The three basic QCs conducted in this study were those used by Lee et al. [9] which consist of a physical limit test, time consistency test, and persistence test. Firstly, the physical limit test determines a value as an error if the measured value is greater than the maximum value or lower than the minimum value. The time consistency test determines an error if the difference between current IoT sensor measurements and the data from a minute prior is greater than the threshold. Lastly, the persistence test determines an error if the size of value fluctuations does not reach the reference size within 60 minutes. We applied these tests to filter the data that are definitely false to increase the possibility that the filtered data are true. However, the filtered data may still contain noisy data. There is no ground truth information, but through these tests, our experiments were conducted on the filtered data regarded as to be possibly true. It is expected that if the filtered data are overall true, our results would be reliable. The normal range reference values for each weather element of basic QC are shown in Table 1. Furthermore, the results of error detection by sequential application of basic QC on collected data are shown in Table 2.

### 2.3. Interpolation of Input Data

Data that was determined to be abnormal by basic QC undergo interpolation to be used as training input data for machine learning. If the data determined to be an error by basic QC are not consecutive and each of them is not at the beginning or the end of training input data of length 10, each error is corrected by linear interpolation regardless of the number of errors. On the contrary, if two consecutive data points at the beginning or end require correction, the entire dataset for that time period is excluded. Interpolation is finished by excluding all data for a given time step if the interpolated data from this process is determined to be an error. Through separate experimentation, we compared the performances of machine learning using data interpolated in this manner against machine learning using noninterpolated data.

## 3. Machine Learning-Based Quality Control

### 3.1. Machine Learning Methods

In this study, primary machine learning algorithms such as SVR, DT, and MLP are used to conduct machine learning-based QC. SVR is a useful technique in the machine learning field that uses SVM [19, 20] to perform regression [21]. SVR is a statistical technique which creates a regression function from training data. Another machine learning algorithm we used is DT. This algorithm uses the decision tree as a prediction model, which allows a clear visual method of identifying the decision-making process. Finally, MLP [22] is a typical machine learning algorithm with a multilayer neural network structure which is used as much as SVR and DT. This algorithm utilizes backpropagation [23] to classify instances as it is a neural network with at least one hidden layer existing between the input and output layers.

We performed MLQC-HT, which independently trains each weather element via SVR, DT, and MLP and analyzed its results. Based on the performance from analyzed results, SVR was selected and applied to MLQC-NT and MLQC-ST as it was considered to be the most meaningful of the three machine learning algorithms. Detailed descriptions of the three QC techniques based on machine learning are given in Section 3.2.

### 3.2. Machine Learning-Based Quality Control

We propose three machine learning-based QC techniques depending on the spatiotemporal characteristics of the input data used for training.

#### 3.2.1. Machine Learning-Based Quality Control Using Homogeneous Temporal Data

**Figure 2:**
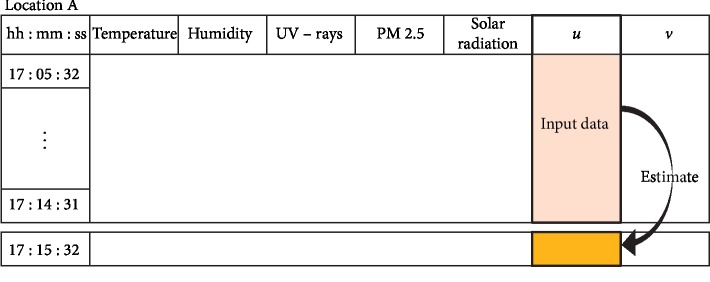
Depiction for data estimation in QC using homogeneous temporal data.

MLQC-HT is a technique that uses past data observations of a single weather element to train for QC. We perform basic QC on the past 10 minutes of independently observed data from each weather element, and then apply linear interpolation on data determined to be errors and use them as training input data to generate the model as shown in Figure 2. Models generated by machine learning are evaluated through 10-fold cross-validation [24]. Based on the model generated by machine learning from the past 10 minutes, the current estimate of each weather element is computed independently. Subsequently, the standard deviation (*σ*) of data from the past 10 minutes is calculated to set the range for passing the machine learning-based QC. Following the method of Lee et al. [7], we set the error range for passing the machine learning-based QC as equation (2). If an estimate produced using machine learning falls within this range, it is deemed *normal*, and if it is out of range, it is deemed as an *error*:

(2) $$\begin{array}{c} \text{estimate}-3\sigma <\text{observed data}<\text{estimate}+3\sigma . \end{array}$$

#### 3.2.2. Machine Learning-Based Quality Control Using Nonhomogeneous Temporal Data

**Figure 3:**
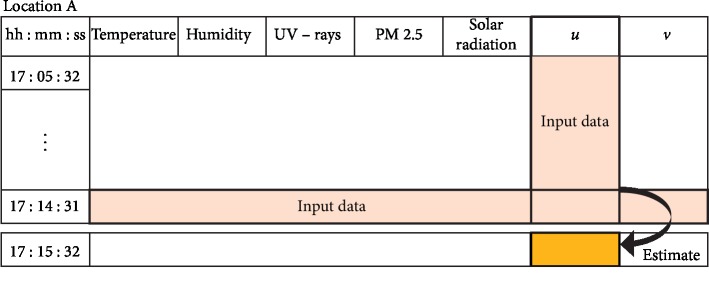
Depiction for data estimation in QC using nonhomogeneous temporal data estimation.

MLQC-NT is a technique for QC of weather elements by training with data from multiple different types of weather elements. For the QC of each weather element, we used the past 10 minutes of data from the same weather element and from other types of weather elements as training input data. After basic QC of the current data from other types of weather elements and the past 10 minutes of data from the selected weather element, data determined as an error are linearly interpolated and used as training data to produce the model, as shown in Figure 3. This QC method also has a model generated by machine learning evaluated through 10-fold cross-validation. Additionally, the current estimate of each weather element is calculated through the model generated by machine learning. Furthermore, the passing range for machine learning-based QC is set by calculating the standard deviation (*σ*) of the training data from the relevant weather element (equation (2)). If an estimate produced by machine learning falls within the set range, it is deemed *normal*, and if it is out of this range, it is deemed as an *error*.

#### 3.2.3. Machine Learning-Based Quality Control Using Spatiotemporal Data

**Figure 4:**
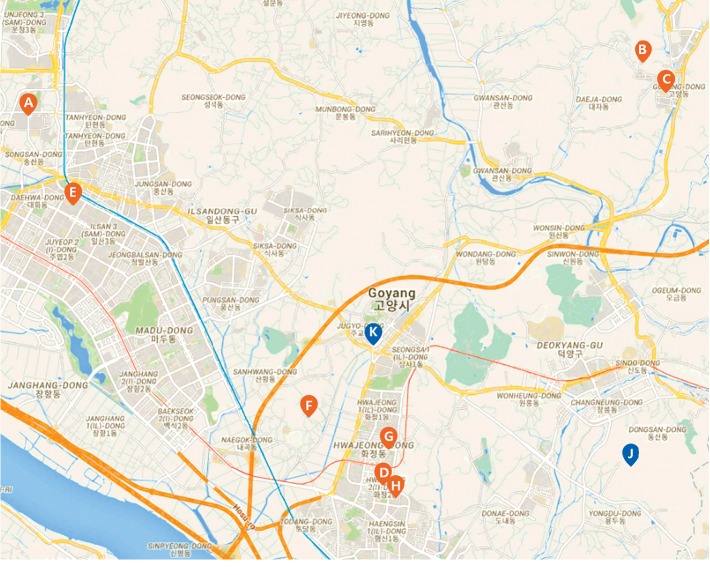
Map showing the location of installed sensors (“A”–“H”: IoT sensor data; “J” and “K”: AWS data) from Google Maps.

**Figure 5:**
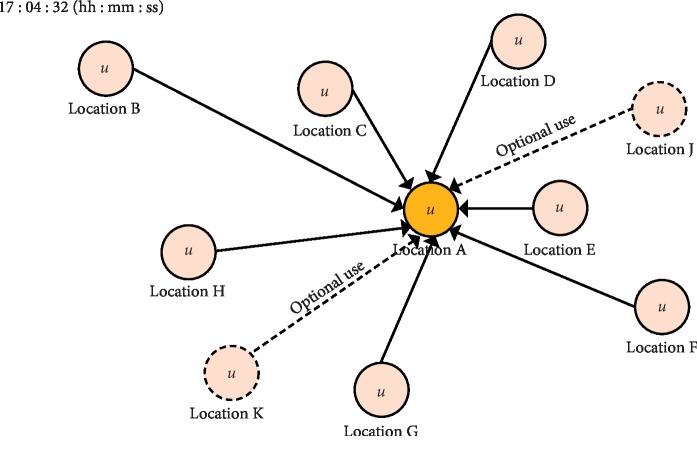
Depiction for data estimation in QC using spatiotemporal data estimation.

MLQC-ST is a QC that is performed with data from sensors in separate locations. This is conducted independently of MLQC-HT and MLQC-NT. MLQC-ST is a technique of finding errors by securing the data that are true from the spatial point of view. In addition, approximation to find errors from the spatial standpoint is more difficult than that from the temporal standpoint. For the testing of this method, we collected data from eight locations in Goyang-si, Gyeonggi-do, South Korea (see Table 3 for detailed information), and used the same time step atmospheric data from each point as training input data. The locations of sensors we installed for data collection are shown in Figure 4 and are labelled “A”–“H”. “J” and “K” are AWSs, and the data acquired from these were optionally used as input data for the given QC technique. After basic QC and linear interpolation of errors, data collected from each point are used as training data to generate a model as shown in Figure 5. This QC technique also undergoes 10-fold cross-validation for evaluation. The current estimate of each weather element is calculated through the model generated by machine learning. In addition, the standard deviation (*σ*) of the trained data is calculated to set the passing range for machine learning QC (equation (2)). If an estimate from machine learning is within the set range, it is deemed *normal*, and if it is outside this range, it is deemed as an *error*.

## 4. Results

We configured the experiment environment with an AMD Ryzen 5 1600X CPU (six-core) at 3.60 GHz and 16 GB of memory. We also used the Waikato Environment for Knowledge Analysis (WEKA) package [25, 26] to implement the three QC techniques and evaluated them through 10-fold cross-validation. Because the performance of 10-fold cross-validation is generally checked with the average value [27–31], we showed the average RMSE for 10 folds. The parameters applied to each machine learning technique are as follows. The training input data for SVR were normalized between 0.0 and 1.0 with the use of the polynomial kernel [32]. In addition, the sequential minimal optimization algorithm [33] was used as the optimization algorithm for parameters of regression. The input neurons of MLP vary by QC techniques, with 10 in MLQC-HT and 16 in MLQC-NT. The MLQC-ST has 7 input neurons by default, and optionally 9, in the case of using additional AWS data. MLP was trained with one hidden layer, 5 hidden neurons, one output neuron, a learning rate of 0.3, and an epoch of 500. Sigmoid was used as an activation function. Finally, the best first search was used for DT. The performance of the combination used in the DT was evaluated based on accuracy and RMSE [34]. In DT, the entropy is calculated to have a value between 0 and 1 by applying the logarithm to the inclusion rate of each class value and adding all the values. The performance of the three QC methods proposed in this paper was evaluated based on RMSE. Furthermore, the correlation between actual observations and the values predicted by the machine learning-generated model can be obtained through the Pearson correlation coefficient.

Firstly, MLQC-HT is a method of independently performing QC to each weather element and its results are shown in Table 4. On average, the RMSE of interpolated data was 39% lower than that of raw data. Among the three machine learning methods, SVR had better RMSE than the other machine learning algorithms for all weather elements excluding solar radiation. From these machine learning algorithms, SVR showed an RMSE value of 11% lower than MLP and 32% lower than DT when averaging QC of interpolated training data and QC of raw data training together. For this reason, SVR was deemed to have the most meaningful results; thus the machine learning method for MLQC-NT and MLQC-ST was set to SVR.

MLQC-NT is a method of using not just the given weather element, but also other associated weather elements to perform QC. Its results are shown in Table 5. Similar to MLQC-HT, this method also conducted separate experiments for training with data which applied interpolation and basic QC and for training with raw data. When QC was performed on raw data with this method, the RMSE was 0.16% lower than when QC was performed on raw data with the SVR utilizing MLQC-HT. Additionally, QC of interpolated data showed a 0.11% lower RMSE for the MLQC-NT method. Through this, it was verified that machine learning-based QC performed with other weather elements in conjunction is better than performing machine learning-based QC on weather elements independently.

MLQC-ST is a QC method which applies the spatiotemporal characteristics of the observation data and operates independently to MLQC-HT and MLQC-NT. In other words, the RMSE values in MLQC-HT and MLQC-NT and those in MLQC-ST cannot be compared. In this method, QC is performed by correlating each weather element data from IoT sensors installed at each of the 8 locations in Goyang-si. All data used in this QC method were normally collected at the eight locations. In other words, data were excluded if they were omitted from any point. Furthermore, additional QC was performed by using AWS data with the atmospheric data from external IoT sensors. As usable AWS data consisted of temperature, humidity, wind direction, and wind speed, tests were performed on a total of four weather elements consisting of temperature, humidity, *u*, and *v*. The averaged results of the 8 locations with MLQC-ST performed are shown in Table 6, and QC conducted with AWS data is shown in Table 7. When QC was performed with AWS data, the RMSE of temperature, humidity, *u*, and *v* was 17% lower on average than if QC was performed only on raw data.

**Figure 6:**
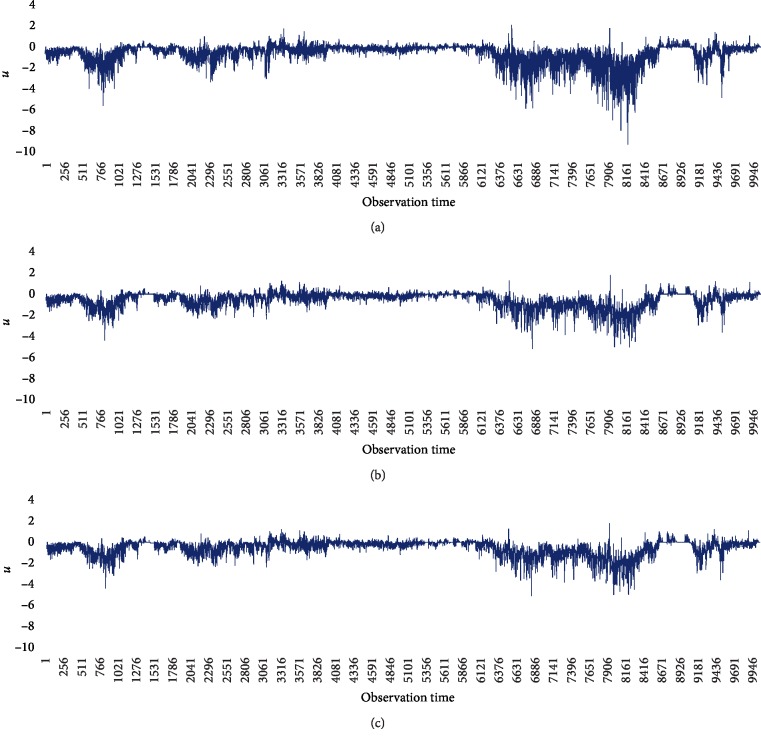
Graphs of *u* data. (a) Raw data of *u*. (b) Corrected data of *u* using MLQC-HT. (c) Corrected data of *u* using MLQC-NT.

**Figure 7:**
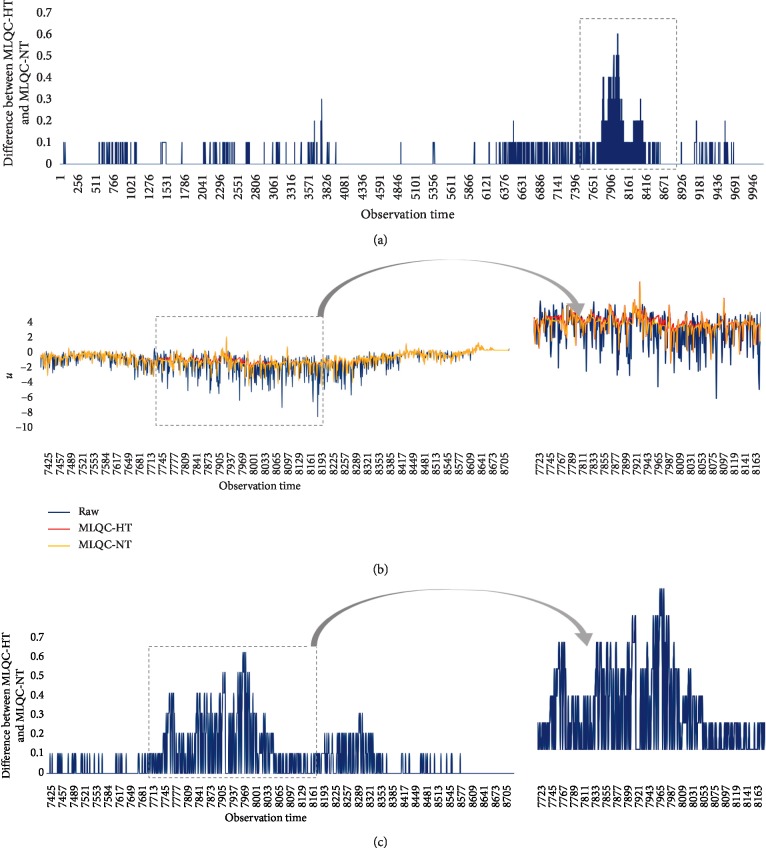
Comparison among raw data and two corrected data (MLQC-HT and MLQC-NT). (a) Visualizing the gap between MLQC-HT and MLQC-NT (the whole time period). (b) Comparison of three types of data (extracted time period). (c) Visualizing the difference between MLQC-HT and MLQC-NT (extracted time period).

We also made estimates based on MLQC-HT and MLQC-NT as the final normal data having undergone the entire process of basic QC, MLQC-HT, MLQC-NT, and MLQC-ST. The results of estimation from the two machine learning-based QC methods are as shown in Table 8. Results of QC performed on the final normal data via MLQC-HT and MLQC-NT methods gave an average RMSE of 0.8990 and 0.8971, respectively, for all weather elements. Through this, it was found that MLQC-NT estimations have better performance than estimations from MLQC-HT. In addition, it was also found that excluding UV-rays, all atmospheric data estimations made using final normal data from undergoing sequential QC had higher accuracy results than estimates made with errors included. The estimated data can be seen in Figure 6 which plots *u* data as a graph. The raw data can be seen plotted as in Figure 6(a).  Figure 6(b) shows data estimated by MLQC-HT, and Figure 6(c) shows estimations made by MLQC-NT. For better comparison, Figure 7(a) shows the difference between MLQC-HT and MLQC-NT for the whole time period.  Figure 7(b) shows an overlay plot and extracts areas showing distinct differences.  Figure 7(c) shows the difference between MLQC-NT and MLQC-HT in the extracted areas. Through this figure, it is possible to see the data estimated using machine learning-based QC and to verify that MLQC-NT is capable of more accurate estimates than MLQC-HT.

## 5. Conclusion

Despite the active research of spatial QC and correction using machine learning in recent times, this study is the first attempt where machine learning-based spatiotemporal QC was performed on multipoint time series data. In this study, three machine learning-based approaches were proposed to perform QC on atmospheric data according to its spatiotemporal characteristics. In addition, QC methods were constructed to suit the type of training data and RMSE was used as an indicator for comparing the performance of the three QC methods. Overall, it was confirmed that machine learning-based QC trained with linear interpolated data had better performance. Comparing the three machine learning-based QC methods discussed in this study, MLQC-NT, which performed machine learning-based QC with other types of weather element in conjunction, was found to have superior performance than MLQC-HT which performed machine learning-based QC on each weather element independently. In addition, MLQC-ST, which was conducted independently of the two previous methods, showed improved performance for temperature, humidity, *u*, and *v* when QC was performed together with AWS data included, rather than with only raw data. Furthermore, when the final normal data, which had undergone basic QC and the three machine learning-based QC methods, were estimated with MLQC-HT and MLQC-NT, the RMSE of MLQC-NT-based estimates was lower. Overall, estimations made with the final normal data, which has undergone basic QC and the three machine learning-based QC methods proposed by this study, showed superior performance to estimations made with error data included.

In future studies, it is anticipated that QC and correction using machine learning will have further improved performance by understanding relationships with other data through methods such as a dimensional reduction technique [35–37]. Furthermore, this study may become the basis of leading to practical studies such as the valuation of collected data and prediction of sensor malfunction.

## Figures and Tables

**Table 1:** Information on sensors collecting weather data and details of basic quality control.

Weather element	Type of sensor	Physical limit [7]		Time consistency	Persistence
		Min	Max	Max variation	Min variation
Temperature (°C)	MHT-100T	−40	60	0.1	0.1
Humidity (%)	MHT-100T	0	100	1	1
UV-rays (W/m^2^)	SU-100	—	—	—	—
Air-PM2.5 (*μ*/m^3^)	ES-642	0.1	359.9	—	—
Solar radiation (W/m^2^)	SP-110	0	1500	—	—
Wind direction (degree)	FST200-202	0	360	—	—
Wind speed (m/s)	FST200-201	0	75	1	0.5

**Table 2:** Results of basic quality control.

Weather element	Physical limit		Time consistency		Persistence	
	#err	Rate_normal_ (%)	#err	Rate_normal_ (%)	#err	Rate_normal_ (%)
Temperature	0	100	251	97.50	13	99.87
Humidity	0	100	798	92.07	2475	75.40
UV-rays	0	100	0	100	0	100
PM2.5	34	99.66	0	100	0	100
Solar radiation	0	100	0	100	0	100
Wind direction	0	100	0	100	0	100
Wind speed	0	100	1571	84.38	402	96.00

^*∗*^The number of weather data is 10,050.

**Table 3:** Location information of sensors used for data collection.

Location	Geographical coordinates (latitude, longitude, altitude)
A	37.6983, 126.7524, 22 m
B	37.7078, 126.8951, 127 m
C	37.7025, 126.9011, 60 m
D	37.6305, 126.8353, 19 m
E	37.6819, 126.7632, 25 m
F	37.6426, 126.8175, 25 m
G	37.6371, 126.8364, 22 m
H	37.6283, 126.8376, 34 m
J	37.6343, 126.8917, 26 m
K	37.6555, 126.8334, 16 m

**Table 4:** Comparison of MLQC-HT results on raw data and interpolated data.

Method	Weather element	Raw data					Interpolated data				
		Correlation coefficient	RMSE	3*σ* error		Time (sec)	Correlation coefficient	RMSE	3*σ* error		Time (sec)
				#err/#total	Rate_err_(%)				#err/#total	Rate_err_(%)	
SVR	Temperature	1.000	0.0589	292/10050	2.91	1464	1.000	**0.0556**	287/9665	2.97	1402
	Humidity	0.9995	0.6456	148/10050	1.47	2378	0.9997	**0.4712**	129/6429	2.01	798
	UV-rays	0.9919	1.7307	4508/10050	44.86	2202	0.9919	**1.7307**	4508/10050	44.86	2202
	PM2.5	0.9209	8.1594	390/10050	3.88	888	0.9834	**3.6107**	388/10013	3.87	928
	Solar radiation	0.9062	0.0049	138/10050	1.37	1126	0.9062	0.0049	138/10050	1.37	1126
	*u*	0.7576	0.5815	432/10050	4.30	717	0.7739	**0.3589**	332/7254	4.58	245
	*v*	0.7639	0.7385	565/10050	5.62	704	0.7837	**0.4251**	436/7254	6.01	219

MLP	Temperature	0.9999	0.0662	1710/10050	17.01	53	0.9999	0.0648	493/9665	5.10	46
	Humidity	0.9993	0.7906	1585/10050	15.77	53	0.9996	0.5484	281/6429	4.37	31
	UV-rays	0.9902	1.9707	3688/10050	36.70	49	0.9902	1.9707	3688/10050	36.70	49
	PM2.5	0.9039	8.9938	772/10050	7.68	54	0.9805	3.9132	405/10013	4.04	50
	Solar radiation	0.9016	0.0051	88/10050	0.88	50	0.9016	0.0051	88/10050	0.88	50
	*u*	0.6273	0.7208	1006/10050	10.01	52	0.654	0.4535	419/7254	5.78	34
	*v*	0.7256	0.7901	1420/10050	14.13	53	0.7075	0.4935	478/7254	6.59	16

DT	Temperature	0.9943	0.6297	7680/10050	76.42	4	0.9945	0.6095	7716/9665	79.83	2
	Humidity	0.9947	2.1775	6752/10050	67.18	5	0.9937	2.2225	4315/6429	67.12	1
	UV-rays	0.9796	2.7371	7033/10050	69.98	2	0.9796	2.7371	7033/10050	69.98	2
	PM2.5	0.8968	9.2503	3466/10050	34.49	8	0.9704	4.7958	3398/10013	33.94	2
	Solar radiation	0.9181	**0.0045**	136/10050	1.35	2	0.9181	**0.0045**	136/10050	1.35	2
	*u*	0.6854	0.6418	794/10050	7.90	5	0.7202	0.391	501/7254	6.91	2
	*v*	0.7130	0.7959	1158/10050	11.52	4	0.7337	0.464	613/7254	8.45	2

^*∗*^SVR is support vector regression, MLP is multilayer perceptron, and DT is decision table.

**Table 5:** Comparison of MLQC-NT results on raw data and interpolated data using SVR.

Weather element	Raw data					Interpolated data				
	Correlation coefficient	RMSE	3*σ* error		Time (sec)	Correlation coefficient	RMSE	3*σ* error		Time (sec)
			#err/#total	Rate_err_(%)				#err/#total	Rate_err_(%)	
Temperature	1.0000	0.0586	177/10050	1.76	1363	1.0000	**0.0553**	192/9665	1.99	1487
Humidity	0.9995	0.6424	504/10050	5.01	6881	0.9997	**0.4690**	185/6429	2.88	3298
UV-rays	0.9919	**1.7307**	4039/10050	40.19	5995	0.9919	**1.7307**	4039/10050	40.19	5995
PM2.5	0.9211	8.1466	382/10050	3.80	5400	0.9834	**3.6097**	372/10013	3.72	3044
Solar radiation	0.9388	**0.0039**	52/10050	0.52	3605	0.9388	**0.0039**	52/10050	0.52	3605
*u*	0.7592	0.5801	560/10050	5.57	1414	0.7772	**0.3563**	386/7254	5.32	543
*v*	0.7650	0.7378	591/10050	5.88	1496	0.7848	**0.4244**	451/7254	6.22	580

**Table 6:** MLQC-ST results on raw data using SVR.

Weather element	Correlation coefficient	RMSE	3*σ* error		Time (sec)
			#err	Rate_err_(%)	
Temperature	0.9968	0.4408	101	1.02	1037.25
Humidity	0.9943	2.1522	135	1.36	1156.625
UV-rays	0.9281	4.6200	1815	18.33	1302.25
PM2.5	0.8982	9.6520	393	3.97	578.5
Solar radiation	0.8122	0.0072	10	0.10	83.125
*u*	0.3927	0.5180	764	7.72	541.75
*v*	0.4869	0.6256	554	5.60	563.25

^*∗*^The number of total weather data is 9,900.

**Table 7:** MLQC-ST results on raw data together with AWS data using SVR.

Weather element	Correlation coefficient	RMSE	3*σ* error		# omitted data	Time (sec)
			#err/#total	Rate_err_(%)		
Temperature	0.9975	0.3863	19/7923	0.24	1742	1048.5
Humidity	0.9927	1.8442	20/4406	0.45	2023	121.375
*u*	0.3659	0.3750	15/2029	0.74	5225	10.5
*v*	0.5347	0.4845	28/2029	1.38	5225	9.875

**Table 8:** Estimated results from the final normal data using SVR.

Weather element	#data	Average	MLQC-HT			MLQC-NT		
			Correlation coefficient	RMSE	Time (sec)	Correlation coefficient	RMSE	Time (sec)
Temperature	9346	8.5291 (5.9146)	1.0000	0.0516	1376	1.0000	**0.0514**	4111
Humidity	6229	77.0694 (21.1415)	0.9998	0.4256	1043	0.9998	**0.4240**	2522
UV-rays	5432	8.4278 (13.6254)	0.9909	2.0303	583	0.9910	**2.0243**	1337
PM2.5	9613	21.8624 (20.9066)	0.9877	3.0947	1366	0.9877	**3.0933**	2709
Solar radiation	9896	0.0064 (0.0114)	0.9176	0.0045	1244	0.9484	**0.0036**	1518
*u*	6844	−0.6097 (0.8803)	0.8066	0.3117	255	0.8101	**0.3089**	360
*v*	6788	−0.2789 (1.1347)	0.8102	0.3751	308	0.8110	**0.3746**	493
Average	—	—	0.9304	0.8990	—	0.9354	**0.8971**	—

^*∗*^The numbers in parentheses are standard deviation.

## Data Availability

The data used to support the findings of this study are available from the corresponding author upon request.
